# 5,7,7,12,14,14-Hexamethyl-4,11-diaza-1,8-diazo­niacyclo­tetra­decane bis­(perchlorate) monohydrate

**DOI:** 10.1107/S1600536812012135

**Published:** 2012-03-24

**Authors:** Saroj K. S. Hazari, Tapashi G. Roy, Babul Chandra Nath, Prashun G. Roy, Seik Weng Ng, Edward R. T. Tiekink

**Affiliations:** a Department of Chemistry, University of Chittagong, Chittagong 4331, Bangladesh; b Department of Chemistry, University of Malaya, 50603 Kuala Lumpur, Malaysia; c Chemistry Department, Faculty of Science, King Abdulaziz University, PO Box 80203 Jeddah, Saudi Arabia

## Abstract

In the title hydrated salt, C_16_H_38_N_4_^2+^·2ClO_4_^−^·H_2_O, the dication is protonated at the diagonally opposite N atoms proximate to the –C(CH_3_)_2_– groups. Within the cavity, there are two ammonium–amine N—H⋯N hydrogen bonds. Supra­molecular layers are formed in the crystal packing whereby the water mol­ecule links two perchlorate anions, and the resultant aggregates are connected to the dications *via* N—H⋯O hydrogen bonds. Layers, with an undulating topology, stack along the *a* axis being connected by C—H⋯O inter­actions.

## Related literature

For background to macrocyclic complexes, see: Hazari *et al.* (2010 ▶). For related structures, see: Hazari *et al.* (2008 ▶). For the synthesis of the macrocyclic ligand, see: Busch *et al.* (1971 ▶).
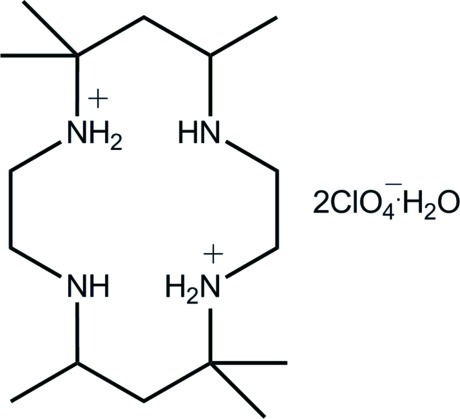


## Experimental

### 

#### Crystal data

C_16_H_38_N_4_^2+^·2ClO_4_^−^·H_2_O
*M* *_r_* = 503.42
Monoclinic, 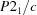

*a* = 11.0930 (1) Å
*b* = 8.7946 (1) Å
*c* = 25.3692 (3) Å
β = 98.435 (1)°
*V* = 2448.21 (5) Å^3^
*Z* = 4
Cu *K*α radiation
μ = 2.84 mm^−1^
*T* = 100 K
0.40 × 0.35 × 0.30 mm

#### Data collection

Agilent SuperNova Dual diffractometer with an Atlas detector
Absorption correction: multi-scan (*CrysAlis PRO*; Agilent, 2011 ▶) *T*_min_ = 0.695, *T*_max_ = 1.000
9569 measured reflections
5000 independent reflections
4665 reflections with *I* > 2σ(*I*)
*R*_int_ = 0.017

#### Refinement

*R*[*F* ^2^ > 2σ(*F* ^2^)] = 0.038
*wR*(*F* ^2^) = 0.105
*S* = 1.03
5000 reflections
312 parameters
8 restraints
H atoms treated by a mixture of independent and constrained refinement
Δρ_max_ = 0.61 e Å^−3^
Δρ_min_ = −0.56 e Å^−3^

### 

Data collection: *CrysAlis PRO* (Agilent, 2011 ▶); cell refinement: *CrysAlis PRO*; data reduction: *CrysAlis PRO*; program(s) used to solve structure: *SHELXS97* (Sheldrick, 2008 ▶); program(s) used to refine structure: *SHELXL97* (Sheldrick, 2008 ▶); molecular graphics: *ORTEP-3* (Farrugia, 1997 ▶) and *DIAMOND* (Brandenburg, 2006 ▶); software used to prepare material for publication: *publCIF* (Westrip, 2010 ▶).

## Supplementary Material

Crystal structure: contains datablock(s) global, I. DOI: 10.1107/S1600536812012135/hg5197sup1.cif

Structure factors: contains datablock(s) I. DOI: 10.1107/S1600536812012135/hg5197Isup2.hkl

Supplementary material file. DOI: 10.1107/S1600536812012135/hg5197Isup3.cml

Additional supplementary materials:  crystallographic information; 3D view; checkCIF report

## Figures and Tables

**Table 1:** Hydrogen-bond geometry (Å, °)

*D*—H⋯*A*	*D*—H	H⋯*A*	*D*⋯*A*	*D*—H⋯*A*
N2—H22⋯N1	0.88 (1)	2.06 (2)	2.8300 (18)	145 (2)
N4—H42⋯N3	0.89 (1)	1.98 (2)	2.7564 (19)	145 (2)
N2—H21⋯O1*w*	0.88 (1)	2.05 (1)	2.8595 (19)	154 (2)
N3—H3⋯O5	0.87 (1)	2.28 (1)	3.1493 (18)	172 (2)
N4—H41⋯O6^i^	0.88 (1)	2.18 (1)	2.9820 (19)	151 (2)
O1*w*—H1*w*⋯O1	0.85 (1)	2.13 (1)	2.9775 (19)	173 (3)
O1*w*—H2*w*⋯O2^ii^	0.85 (1)	1.98 (1)	2.827 (2)	177 (4)
C8—H8*B*⋯O4^iii^	0.99	2.44	3.179 (2)	131

Symmetry codes: (i) 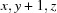; (ii) 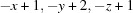; (iii) 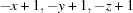.

**Fig. 1.**
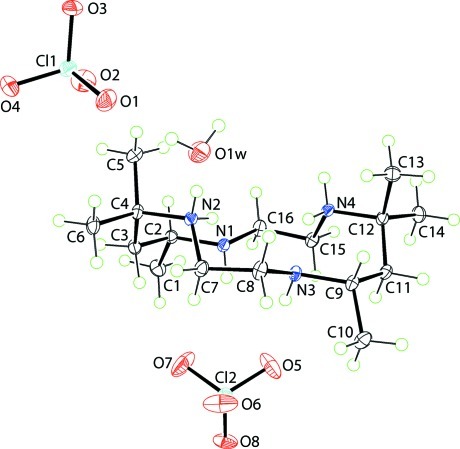
The molecular structure of the constituents of (I) showing the atom-labelling scheme and displacement ellipsoids at the 50% probability level.

**Fig. 2.**
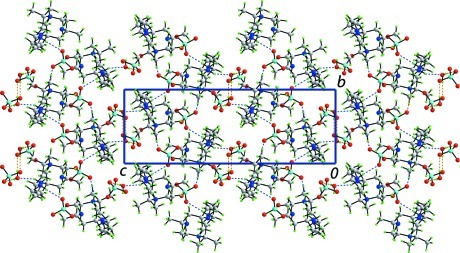
A view of the supramolecular layer in the bc plane in (I). The O—H···O and N—H···O hydrogen bonds are shown as orange and blue dashed lines, respectively.

**Fig. 3.**
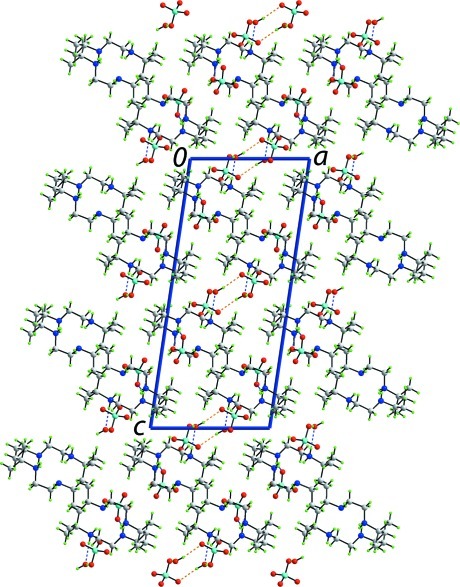
A view of the unit-cell contents in projection down the b axis in (I). The O—H···O and N—H···O hydrogen bonds are shown as orange and blue dashed lines, respectively.

## supplementary crystallographic information

### Comment

As a continuation of systematic studies into the synthesis, characterization and biological activities of substituted tetraazamacrocyclic ligands and their metal complexes (Hazari *et al.*, 2008; Hazari *et al.*, 2010), crystals of the title hydrated salt, (I), were isolated and characterized crystallographically.

In (I), Fig. 1, the crystallographic asymmetric unit comprises a dipositive cation with the charge balance provided by two ClO_4_^-^ ions, and is completed by a water molecule of hydration. Crystallography shows that protonation has occurred at diagonally opposite N atoms that are proximate to the C atom carrying two methyl substituents. A similar pattern of protonation was observed in the octa-methyl analogues characterized as the nitrate and acetate salts, the latter as a trihydrate (Hazari *et al.*, 2008). Within the cavity, there are two N—H···N hydrogen bonds where the donor hydrogen is bound to an ammonium centre, Table 1. Three of the remaining N—H atoms form hydrogen bonding interactions to the water molecule or perchlorate-O atoms, Table 1, leaving one N—H hydrogen atom not involved in a significant intermolecular contact. The water molecule forms two donor interactions with perchlorate-O atoms derived from different perchlorate anions. The hydrogen bonding interactions lead to the formation of supramolecular layers in the *bc* plane, Fig. 2. The layers have a zigzag topology and stack along the *a* axis with the major interactions between them being of the type C—H···O, Fig. 3 and Table 1.

### Experimental

The compound 5,7,7,12,14,14-hexamethyl-1,4,8,11-tetraazacyclotetra-1,8-decadiene.2HClO_4_, prepared by a modified method from the reported method (Busch *et al.*, 1971), on reduction with NaBH_4_, yields an isomeric mixture of saturated macrocycles, Me_6_[14]anes, which have been resolved into two distinct isomers namely 'tet a' and 'tet b'. During the synthesis of dihydrotrifluroacetate salt of 'tet b', by the reaction of 'tet b' with trifluoroacetic acid, crystals were formed from the mother liquor by slow evaporation. Yield 55%. *M*.pt: 523–525 K. Anal. Calc. for C_16_H_40_N_4_Cl_2_O_9_, C, 38.17; H, 8.01; N, 11.13%. Found: C, 38.15; H, 8.09; N, 10.98%. FT—IR (KBr, cm^-1^) 3210 ν(N—H), 2975 ν(C—H), 1372 ν(CH_3_), 1184 ν(C—C), 1125, 623 ν(ClO_4_). The same product was isolated during the attempted synthesis of the *N*-pendent ligand of 'tet b' by the reaction of 'tet b' with allyl chloride. The formation of the unexpected perchlorate salt of the compound may be due to presence HClO_4_ in trifluoroacetic acid solution as well in the allyl chloride solution, respectively.

### Refinement

The C-bound H-atoms were placed in calculated positions (C—H = 0.98–1.00 Å) and were included in the refinement in the riding model approximation, with *U*_iso_(H) = 1.2–1.5*U*_equiv_(N,*C*). The O—H and N—H atoms were located from a difference map and refined with O—H = 0.84±0.01 Å and N—H = 0.88±0.01 Å, respectively, and with *U*_iso_(H)= 1.5*U*_equiv_(O) or 1.2*U*_equiv_(N).

### Crystal data

**Table:**

C_16_H_38_N_4_^2+^·2ClO_4_^−^·H_2_O	*F*(000) = 1080
*M**_r_* = 503.42	*D*_x_ = 1.366 Mg m^−^^3^
Monoclinic, *P*2_1_/*c*	Cu *K*α radiation, λ = 1.54184 Å
Hall symbol: -P 2ybc	Cell parameters from 6194 reflections
*a* = 11.0930 (1) Å	θ = 3.5–76.3°
*b* = 8.7946 (1) Å	µ = 2.84 mm^−^^1^
*c* = 25.3692 (3) Å	*T* = 100 K
β = 98.435 (1)°	Block, colourless
*V* = 2448.21 (5) Å^3^	0.40 × 0.35 × 0.30 mm
*Z* = 4	

### Data collection

**Table:**

Agilent SuperNova Dual diffractometer with an Atlas detector	5000 independent reflections
Radiation source: SuperNova (Cu) X-ray Source	4665 reflections with *I* > 2σ(*I*)
Mirror monochromator	*R*_int_ = 0.017
Detector resolution: 10.4041 pixels mm^-1^	θ_max_ = 76.5°, θ_min_ = 3.5°
ω scan	*h* = −13→13
Absorption correction: multi-scan (*CrysAlis PRO*; Agilent, 2011)	*k* = −10→6
*T*_min_ = 0.695, *T*_max_ = 1.000	*l* = −29→31
9569 measured reflections	

### Refinement

**Table:**

Refinement on *F*^2^	Primary atom site location: structure-invariant direct methods
Least-squares matrix: full	Secondary atom site location: difference Fourier map
*R*[*F*^2^ > 2σ(*F*^2^)] = 0.038	Hydrogen site location: inferred from neighbouring sites
*wR*(*F*^2^) = 0.105	H atoms treated by a mixture of independent and constrained refinement
*S* = 1.03	*w* = 1/[σ^2^(*F*_o_^2^) + (0.0609*P*)^2^ + 1.6737*P*] where *P* = (*F*_o_^2^ + 2*F*_c_^2^)/3
5000 reflections	(Δ/σ)_max_ = 0.001
312 parameters	Δρ_max_ = 0.61 e Å^−^^3^
8 restraints	Δρ_min_ = −0.56 e Å^−^^3^

### Special details

**Table:**

Geometry. All e.s.d.'s (except the e.s.d. in the dihedral angle between two l.s. planes) are estimated using the full covariance matrix. The cell e.s.d.'s are taken into account individually in the estimation of e.s.d.'s in distances, angles and torsion angles; correlations between e.s.d.'s in cell parameters are only used when they are defined by crystal symmetry. An approximate (isotropic) treatment of cell e.s.d.'s is used for estimating e.s.d.'s involving l.s. planes.
Refinement. Refinement of *F*^2^ against ALL reflections. The weighted *R*-factor *wR* and goodness of fit *S* are based on *F*^2^, conventional *R*-factors *R* are based on *F*, with *F* set to zero for negative *F*^2^. The threshold expression of *F*^2^ > σ(*F*^2^) is used only for calculating *R*-factors(gt) *etc*. and is not relevant to the choice of reflections for refinement. *R*-factors based on *F*^2^ are statistically about twice as large as those based on *F*, and *R*- factors based on ALL data will be even larger.

### Fractional atomic coordinates and isotropic or equivalent isotropic displacement parameters (Å^2^)

**Table:**

	*x*	*y*	*z*	*U*_iso_*/*U*_eq_	
Cl1	0.31093 (3)	0.82773 (4)	0.546168 (14)	0.01665 (11)	
Cl2	0.83060 (3)	0.38578 (4)	0.287184 (13)	0.01370 (11)	
O1	0.42268 (11)	0.75538 (15)	0.56951 (5)	0.0268 (3)	
O2	0.32605 (13)	0.88170 (16)	0.49335 (5)	0.0298 (3)	
O3	0.28350 (12)	0.95396 (15)	0.57816 (5)	0.0258 (3)	
O4	0.21214 (11)	0.72094 (15)	0.54079 (5)	0.0261 (3)	
O5	0.90879 (15)	0.51760 (18)	0.29309 (6)	0.0383 (4)	
O6	0.83712 (16)	0.30970 (16)	0.33756 (5)	0.0382 (4)	
O7	0.70924 (13)	0.4375 (2)	0.27069 (7)	0.0451 (4)	
O8	0.86602 (13)	0.28549 (15)	0.24776 (5)	0.0279 (3)	
O1w	0.62426 (13)	0.80297 (17)	0.50563 (5)	0.0276 (3)	
N1	0.69983 (11)	0.81512 (15)	0.29586 (5)	0.0124 (3)	
N2	0.63078 (11)	0.73862 (15)	0.39559 (5)	0.0127 (3)	
N3	0.89992 (11)	0.70336 (15)	0.39858 (5)	0.0124 (3)	
N4	0.91280 (11)	0.98528 (15)	0.35226 (5)	0.0125 (3)	
C1	0.54974 (15)	0.7498 (2)	0.21479 (6)	0.0191 (3)	
H1A	0.5885	0.8231	0.1935	0.029*	
H1B	0.4623	0.7448	0.2016	0.029*	
H1C	0.5863	0.6492	0.2119	0.029*	
C2	0.56883 (13)	0.80012 (18)	0.27322 (6)	0.0133 (3)	
H2A	0.5291	0.9015	0.2754	0.016*	
C3	0.50736 (14)	0.68698 (18)	0.30677 (6)	0.0149 (3)	
H3A	0.5505	0.5885	0.3064	0.018*	
H3B	0.4229	0.6708	0.2889	0.018*	
C4	0.50164 (13)	0.72858 (18)	0.36520 (6)	0.0137 (3)	
C5	0.44573 (14)	0.88612 (19)	0.37008 (7)	0.0176 (3)	
H5A	0.4433	0.9089	0.4077	0.026*	
H5B	0.3627	0.8877	0.3505	0.026*	
H5C	0.4953	0.9627	0.3551	0.026*	
C6	0.42988 (15)	0.6089 (2)	0.39099 (7)	0.0190 (3)	
H6A	0.4267	0.6371	0.4281	0.028*	
H6B	0.4700	0.5099	0.3899	0.028*	
H6C	0.3469	0.6028	0.3716	0.028*	
C7	0.69904 (14)	0.59435 (18)	0.40941 (6)	0.0159 (3)	
H7A	0.7015	0.5338	0.3767	0.019*	
H7B	0.6567	0.5336	0.4340	0.019*	
C8	0.82827 (14)	0.6290 (2)	0.43569 (6)	0.0166 (3)	
H8A	0.8253	0.6961	0.4668	0.020*	
H8B	0.8689	0.5332	0.4487	0.020*	
C9	1.03115 (13)	0.71842 (18)	0.42113 (6)	0.0134 (3)	
H9A	1.0365	0.7608	0.4580	0.016*	
C10	1.09813 (15)	0.5659 (2)	0.42452 (7)	0.0192 (3)	
H10A	1.0580	0.4949	0.4461	0.029*	
H10B	1.1829	0.5809	0.4410	0.029*	
H10C	1.0964	0.5241	0.3886	0.029*	
C11	1.09391 (13)	0.82989 (18)	0.38754 (6)	0.0133 (3)	
H11A	1.0905	0.7862	0.3514	0.016*	
H11B	1.1809	0.8361	0.4032	0.016*	
C12	1.04329 (13)	0.99271 (18)	0.38168 (6)	0.0134 (3)	
C13	1.03266 (15)	1.0660 (2)	0.43525 (6)	0.0190 (3)	
H13A	1.1138	1.0741	0.4564	0.028*	
H13B	0.9803	1.0033	0.4544	0.028*	
H13C	0.9971	1.1677	0.4294	0.028*	
C14	1.12120 (14)	1.09205 (19)	0.35056 (7)	0.0179 (3)	
H14A	1.2049	1.0955	0.3694	0.027*	
H14B	1.0875	1.1952	0.3474	0.027*	
H14C	1.1212	1.0492	0.3149	0.027*	
C15	0.89575 (13)	0.94676 (18)	0.29431 (6)	0.0139 (3)	
H15A	0.9337	0.8470	0.2892	0.017*	
H15B	0.9363	1.0243	0.2747	0.017*	
C16	0.76101 (14)	0.94086 (19)	0.27244 (6)	0.0154 (3)	
H16A	0.7223	1.0381	0.2802	0.019*	
H16B	0.7510	0.9282	0.2333	0.019*	
H1w	0.5629 (18)	0.787 (4)	0.5213 (11)	0.059 (9)*	
H2w	0.641 (3)	0.8975 (15)	0.5067 (16)	0.088 (13)*	
H1	0.7341 (18)	0.7279 (15)	0.2895 (8)	0.019 (5)*	
H3	0.8958 (18)	0.648 (2)	0.3698 (6)	0.018 (5)*	
H22	0.6697 (18)	0.791 (2)	0.3737 (7)	0.026 (6)*	
H21	0.6276 (19)	0.788 (2)	0.4254 (6)	0.023 (5)*	
H41	0.8779 (18)	1.0722 (15)	0.3581 (8)	0.021 (5)*	
H42	0.8796 (19)	0.913 (2)	0.3698 (8)	0.028 (6)*	

### Atomic displacement parameters (Å^2^)

**Table:**

	*U*^11^	*U*^22^	*U*^33^	*U*^12^	*U*^13^	*U*^23^
Cl1	0.01667 (19)	0.0170 (2)	0.01711 (19)	0.00152 (13)	0.00511 (13)	−0.00033 (13)
Cl2	0.01564 (18)	0.01343 (19)	0.01328 (18)	0.00174 (12)	0.00624 (13)	0.00070 (12)
O1	0.0185 (6)	0.0267 (7)	0.0341 (7)	0.0054 (5)	−0.0001 (5)	0.0033 (6)
O2	0.0403 (8)	0.0316 (7)	0.0191 (6)	−0.0007 (6)	0.0099 (5)	0.0011 (5)
O3	0.0273 (6)	0.0213 (6)	0.0316 (7)	−0.0028 (5)	0.0139 (5)	−0.0116 (5)
O4	0.0229 (6)	0.0214 (6)	0.0331 (7)	−0.0055 (5)	0.0014 (5)	−0.0056 (5)
O5	0.0504 (9)	0.0393 (8)	0.0296 (7)	−0.0294 (7)	0.0204 (6)	−0.0167 (6)
O6	0.0747 (11)	0.0252 (7)	0.0196 (7)	0.0184 (7)	0.0227 (7)	0.0106 (5)
O7	0.0206 (7)	0.0663 (11)	0.0464 (9)	0.0191 (7)	−0.0019 (6)	−0.0046 (8)
O8	0.0463 (8)	0.0187 (6)	0.0241 (6)	0.0007 (6)	0.0229 (6)	−0.0047 (5)
O1w	0.0290 (7)	0.0316 (7)	0.0252 (7)	−0.0012 (6)	0.0135 (5)	−0.0016 (6)
N1	0.0106 (6)	0.0129 (6)	0.0140 (6)	0.0004 (5)	0.0024 (5)	0.0012 (5)
N2	0.0113 (6)	0.0142 (6)	0.0131 (6)	−0.0008 (5)	0.0036 (5)	−0.0010 (5)
N3	0.0102 (6)	0.0164 (6)	0.0108 (6)	−0.0011 (5)	0.0023 (5)	0.0007 (5)
N4	0.0107 (6)	0.0132 (6)	0.0142 (6)	0.0010 (5)	0.0042 (5)	0.0002 (5)
C1	0.0224 (8)	0.0215 (8)	0.0130 (7)	−0.0034 (6)	0.0009 (6)	−0.0019 (6)
C2	0.0118 (7)	0.0146 (7)	0.0132 (7)	−0.0004 (5)	0.0014 (5)	−0.0012 (6)
C3	0.0134 (7)	0.0159 (7)	0.0153 (7)	−0.0030 (6)	0.0019 (5)	−0.0015 (6)
C4	0.0094 (6)	0.0166 (8)	0.0153 (7)	−0.0015 (6)	0.0026 (5)	0.0000 (6)
C5	0.0154 (7)	0.0201 (8)	0.0183 (7)	0.0033 (6)	0.0054 (6)	−0.0006 (6)
C6	0.0143 (7)	0.0220 (8)	0.0217 (8)	−0.0048 (6)	0.0064 (6)	0.0012 (6)
C7	0.0129 (7)	0.0148 (7)	0.0206 (8)	0.0000 (6)	0.0041 (6)	0.0047 (6)
C8	0.0132 (7)	0.0221 (8)	0.0147 (7)	−0.0002 (6)	0.0031 (6)	0.0066 (6)
C9	0.0098 (7)	0.0183 (8)	0.0119 (7)	0.0009 (6)	0.0013 (5)	0.0009 (6)
C10	0.0163 (7)	0.0202 (8)	0.0212 (8)	0.0050 (6)	0.0030 (6)	0.0039 (6)
C11	0.0098 (6)	0.0176 (8)	0.0130 (7)	0.0010 (5)	0.0033 (5)	−0.0004 (6)
C12	0.0104 (6)	0.0162 (7)	0.0142 (7)	−0.0008 (6)	0.0034 (5)	−0.0015 (6)
C13	0.0186 (7)	0.0223 (8)	0.0166 (7)	−0.0004 (6)	0.0045 (6)	−0.0059 (6)
C14	0.0150 (7)	0.0181 (8)	0.0218 (8)	−0.0042 (6)	0.0064 (6)	0.0002 (6)
C15	0.0131 (7)	0.0172 (8)	0.0123 (7)	−0.0002 (6)	0.0047 (5)	0.0029 (6)
C16	0.0132 (7)	0.0169 (8)	0.0161 (7)	−0.0004 (6)	0.0014 (5)	0.0061 (6)

### Geometric parameters (Å, º)

**Table:**

Cl1—O3	1.4339 (12)	C4—C5	1.530 (2)
Cl1—O4	1.4345 (13)	C5—H5A	0.9800
Cl1—O1	1.4412 (12)	C5—H5B	0.9800
Cl1—O2	1.4542 (13)	C5—H5C	0.9800
Cl2—O7	1.4245 (14)	C6—H6A	0.9800
Cl2—O8	1.4312 (12)	C6—H6B	0.9800
Cl2—O6	1.4349 (13)	C6—H6C	0.9800
Cl2—O5	1.4425 (14)	C7—C8	1.521 (2)
O1w—H1w	0.848 (10)	C7—H7A	0.9900
O1w—H2w	0.852 (10)	C7—H7B	0.9900
N1—C16	1.4679 (19)	C8—H8A	0.9900
N1—C2	1.4881 (18)	C8—H8B	0.9900
N1—H1	0.882 (9)	C9—C11	1.532 (2)
N2—C7	1.493 (2)	C9—C10	1.530 (2)
N2—C4	1.5266 (18)	C9—H9A	1.0000
N2—H22	0.881 (10)	C10—H10A	0.9800
N2—H21	0.879 (9)	C10—H10B	0.9800
N3—C8	1.4713 (19)	C10—H10C	0.9800
N3—C9	1.4899 (18)	C11—C12	1.537 (2)
N3—H3	0.874 (9)	C11—H11A	0.9900
N4—C15	1.4936 (19)	C11—H11B	0.9900
N4—C12	1.5296 (18)	C12—C13	1.524 (2)
N4—H41	0.879 (9)	C12—C14	1.528 (2)
N4—H42	0.887 (10)	C13—H13A	0.9800
C1—C2	1.532 (2)	C13—H13B	0.9800
C1—H1A	0.9800	C13—H13C	0.9800
C1—H1B	0.9800	C14—H14A	0.9800
C1—H1C	0.9800	C14—H14B	0.9800
C2—C3	1.533 (2)	C14—H14C	0.9800
C2—H2A	1.0000	C15—C16	1.517 (2)
C3—C4	1.537 (2)	C15—H15A	0.9900
C3—H3A	0.9900	C15—H15B	0.9900
C3—H3B	0.9900	C16—H16A	0.9900
C4—C6	1.524 (2)	C16—H16B	0.9900
			
O3—Cl1—O4	109.76 (8)	C4—C6—H6C	109.5
O3—Cl1—O1	110.50 (8)	H6A—C6—H6C	109.5
O4—Cl1—O1	110.43 (8)	H6B—C6—H6C	109.5
O3—Cl1—O2	109.37 (8)	N2—C7—C8	110.22 (13)
O4—Cl1—O2	108.41 (8)	N2—C7—H7A	109.6
O1—Cl1—O2	108.32 (8)	C8—C7—H7A	109.6
O7—Cl2—O8	109.64 (9)	N2—C7—H7B	109.6
O7—Cl2—O6	109.43 (10)	C8—C7—H7B	109.6
O8—Cl2—O6	110.61 (8)	H7A—C7—H7B	108.1
O7—Cl2—O5	107.56 (11)	N3—C8—C7	111.81 (13)
O8—Cl2—O5	110.20 (8)	N3—C8—H8A	109.3
O6—Cl2—O5	109.35 (10)	C7—C8—H8A	109.3
H1w—O1w—H2w	110 (3)	N3—C8—H8B	109.3
C16—N1—C2	113.19 (12)	C7—C8—H8B	109.3
C16—N1—H1	110.1 (14)	H8A—C8—H8B	107.9
C2—N1—H1	105.9 (14)	N3—C9—C11	109.97 (12)
C7—N2—C4	118.40 (12)	N3—C9—C10	112.50 (13)
C7—N2—H22	108.3 (15)	C11—C9—C10	109.63 (12)
C4—N2—H22	102.9 (15)	N3—C9—H9A	108.2
C7—N2—H21	107.7 (14)	C11—C9—H9A	108.2
C4—N2—H21	108.1 (14)	C10—C9—H9A	108.2
H22—N2—H21	111 (2)	C9—C10—H10A	109.5
C8—N3—C9	112.51 (12)	C9—C10—H10B	109.5
C8—N3—H3	108.6 (14)	H10A—C10—H10B	109.5
C9—N3—H3	107.4 (13)	C9—C10—H10C	109.5
C15—N4—C12	117.65 (11)	H10A—C10—H10C	109.5
C15—N4—H41	111.5 (14)	H10B—C10—H10C	109.5
C12—N4—H41	106.9 (14)	C9—C11—C12	117.42 (12)
C15—N4—H42	109.1 (15)	C9—C11—H11A	107.9
C12—N4—H42	102.7 (15)	C12—C11—H11A	107.9
H41—N4—H42	108.4 (19)	C9—C11—H11B	107.9
C2—C1—H1A	109.5	C12—C11—H11B	107.9
C2—C1—H1B	109.5	H11A—C11—H11B	107.2
H1A—C1—H1B	109.5	C13—C12—C14	110.03 (13)
C2—C1—H1C	109.5	C13—C12—N4	105.17 (12)
H1A—C1—H1C	109.5	C14—C12—N4	109.81 (12)
H1B—C1—H1C	109.5	C13—C12—C11	112.50 (13)
N1—C2—C1	112.82 (13)	C14—C12—C11	110.87 (12)
N1—C2—C3	109.39 (12)	N4—C12—C11	108.27 (12)
C1—C2—C3	109.88 (13)	C12—C13—H13A	109.5
N1—C2—H2A	108.2	C12—C13—H13B	109.5
C1—C2—H2A	108.2	H13A—C13—H13B	109.5
C3—C2—H2A	108.2	C12—C13—H13C	109.5
C2—C3—C4	117.75 (13)	H13A—C13—H13C	109.5
C2—C3—H3A	107.9	H13B—C13—H13C	109.5
C4—C3—H3A	107.9	C12—C14—H14A	109.5
C2—C3—H3B	107.9	C12—C14—H14B	109.5
C4—C3—H3B	107.9	H14A—C14—H14B	109.5
H3A—C3—H3B	107.2	C12—C14—H14C	109.5
C6—C4—N2	109.49 (12)	H14A—C14—H14C	109.5
C6—C4—C5	110.30 (13)	H14B—C14—H14C	109.5
N2—C4—C5	105.56 (12)	N4—C15—C16	110.09 (12)
C6—C4—C3	110.35 (13)	N4—C15—H15A	109.6
N2—C4—C3	109.42 (12)	C16—C15—H15A	109.6
C5—C4—C3	111.60 (13)	N4—C15—H15B	109.6
C4—C5—H5A	109.5	C16—C15—H15B	109.6
C4—C5—H5B	109.5	H15A—C15—H15B	108.2
H5A—C5—H5B	109.5	N1—C16—C15	111.53 (12)
C4—C5—H5C	109.5	N1—C16—H16A	109.3
H5A—C5—H5C	109.5	C15—C16—H16A	109.3
H5B—C5—H5C	109.5	N1—C16—H16B	109.3
C4—C6—H6A	109.5	C15—C16—H16B	109.3
C4—C6—H6B	109.5	H16A—C16—H16B	108.0
H6A—C6—H6B	109.5		
			
C16—N1—C2—C1	67.79 (17)	C8—N3—C9—C11	−165.84 (13)
C16—N1—C2—C3	−169.58 (12)	C8—N3—C9—C10	71.65 (16)
N1—C2—C3—C4	63.55 (17)	N3—C9—C11—C12	58.64 (17)
C1—C2—C3—C4	−172.09 (13)	C10—C9—C11—C12	−177.16 (13)
C7—N2—C4—C6	46.82 (17)	C15—N4—C12—C13	168.25 (13)
C7—N2—C4—C5	165.54 (13)	C15—N4—C12—C14	49.90 (17)
C7—N2—C4—C3	−74.24 (16)	C15—N4—C12—C11	−71.28 (16)
C2—C3—C4—C6	175.99 (13)	C9—C11—C12—C13	52.22 (17)
C2—C3—C4—N2	−63.47 (17)	C9—C11—C12—C14	175.91 (13)
C2—C3—C4—C5	52.98 (17)	C9—C11—C12—N4	−63.57 (16)
C4—N2—C7—C8	175.73 (12)	C12—N4—C15—C16	178.58 (12)
C9—N3—C8—C7	−171.92 (13)	C2—N1—C16—C15	−176.80 (12)
N2—C7—C8—N3	−66.08 (17)	N4—C15—C16—N1	−66.07 (17)

### Hydrogen-bond geometry (Å, º)

**Table:**

*D*—H···*A*	*D*—H	H···*A*	*D*···*A*	*D*—H···*A*
N2—H22···N1	0.88 (1)	2.06 (2)	2.8300 (18)	145 (2)
N4—H42···N3	0.89 (1)	1.98 (2)	2.7564 (19)	145 (2)
N2—H21···O1*w*	0.88 (1)	2.05 (1)	2.8595 (19)	154 (2)
N3—H3···O5	0.87 (1)	2.28 (1)	3.1493 (18)	172 (2)
N4—H41···O6^i^	0.88 (1)	2.18 (1)	2.9820 (19)	151 (2)
O1*w*—H1*w*···O1	0.85 (1)	2.13 (1)	2.9775 (19)	173 (3)
O1*w*—H2*w*···O2^ii^	0.85 (1)	1.98 (1)	2.827 (2)	177 (4)
C8—H8*B*···O4^iii^	0.99	2.44	3.179 (2)	131

Symmetry codes: (i) *x*, *y*+1, *z*; (ii) −*x*+1, −*y*+2, −*z*+1; (iii) −*x*+1, −*y*+1, −*z*+1.
